# Evaluation of hepatitis C virus antibody assay using dried blood spot samples

**DOI:** 10.1038/s41598-022-07821-0

**Published:** 2022-03-08

**Authors:** Vera Holzmayer, Russell Taylor, Mary C. Kuhns, Susan H. Gawel, Nicaise Ndembi, Dora Mbanya, Lazare Kaptue, Mary A. Rodgers, Gavin Cloherty

**Affiliations:** 1 Abbott Laboratories, Abbott Diagnostics Division, 100 Abbott Park Road, Bldg. AP20, Abbott Park, IL 60064-3500 USA; 2 Africa Centres for Disease Control and Prevention, Addis Ababa, Ethiopia; 3 Universite de Yaounde I, Yaounde, Cameroon; 4 Universite des Montagnes, Bangangte, Cameroon

**Keywords:** Epidemiology, Viral hepatitis, Laboratory techniques and procedures

## Abstract

Early diagnosis of hepatitis C virus (HCV) infection is essential for prompt initiation of treatment and prevention of transmission, yet several logistical barriers continue to limit access to HCV testing. Dried blood spot (DBS) technology involves a simple fingerstick that eliminates the need for trained personnel, and DBS can be stored and transported at room temperature. We evaluated the use of DBS whole blood samples in the modified Abbott ARCHITECT anti-HCV assay, comparing assay performance against the standard assay run using DBS and venous plasma samples. 144 HCV positive and 104 HCV negative matched venous plasma and whole blood specimens were selected from a retrospective study with convenience sampling in Cameroon. Results obtained using a modified volume DBS assay were highly correlated to the results of the standard assay run with plasma on clinical samples and dilution series (R^2^ = 0.71 and 0.99 respectively). The ARCHITECT Anti-HCV assay with input volume modification more accurately detects HCV antibodies in DBS whole blood samples with 100% sensitivity and specificity, while the standard assay had 90.97% sensitivity. The use of DBS has the potential to expand access to HCV testing to underserved or marginalized populations with limited access to direct HCV care.

## Introduction

Approximately 71 million people worldwide^1^, and 2.4 million in the United States^2^, have chronic hepatitis C virus infection, with an estimated 50,000 new infections in the US in 2018^2^. In Africa, HCV infection affect an estimated 27 million people, with regional prevalence rates ranging from 0.9 to 6.0%^3^. While highly effective direct-acting anti-viral treatments for HCV infection are available^4^, the diagnosis of hepatitis C infection to initiate treatment and prevent transmission remains challenging. Early stages of infection are often asymptomatic and economic and logistical barriers limit access to diagnostic testing. To reduce the health burden of HCV infection and to support the World Health Organization’s (WHO) goal to eliminate viral hepatitis as a public health threat by 2030, there is a need to expand HCV testing, while also balancing sensitivity, specificity, and cost^5^.

In 2017, the WHO recommended the use of dried blood spot (DBS) samples to increase access to HBV and HCV testing^6^. DBS samples are collected by a simple fingerstick, with capillary blood transferred to filter paper, dried, and transported to the central lab, where it is eluted from the filter paper for testing. DBS removes the need for personnel trained in venipuncture and samples can be stored at room temperature. Thus, DBS provides an easy, low-cost option for sample collection in low- and middle-income countries, as well as in low-resource rural areas with limited access to direct HCV testing or health care. DBS samples have been used in HCV antigen testing^7,8^ as well as for HIV, HCV, and HBV antibody testing^9–11^.

While the use of DBS samples would increase the accessibility of HCV testing, there is a need to ensure that test specificity and sensitivity with DBS samples are comparable to those with venous plasma samples. In this study, we examined whether DBS whole blood samples could be used as the sample type for the Abbott ARCHITECT Anti-HCV assay (List 6C37, Abbott GmbH, Wiesbaden, Germany), which detects HCV antibodies in venous serum and plasma. We compared the performance of the current ARCHITECT Anti-HCV assay run with venous plasma samples per the manufacturer’s instructions with the performance of the assay using a modified input volume for DBS samples prepared from venous whole blood.

## Results

### Assay dilutional sensitivity with DBS samples

The dilutional sensitivity of the ARCHITECT Anti-HCV assay with DBS samples was evaluated using dilution series created from two randomly selected plasma samples with high S/CO values. The two plasma samples were diluted in parallel in negative plasma and negative whole blood with a dilution factor ranging from 10 to 1000. The DBS S/CO results were compared to those for each plasma sample at each dilution level (Table 1).

**Table 1:** Dilution sensitivity for 6-mm and 12-mm DBS eluted in EB and AD, tested with the standard and modified sample volume ARCHITECT anti-HCV assay.

	Plasma	12-mm DBS, STD assay, EB	12-mm DBS, STD assay, AD	12-mm DBS, MSV assay, EB	12-mm DBS, MSV assay, AD	6-mm DBS, MSV assay, EB	6-mm DBS, MSV assay, AD
DF	S/CO	S/CO	S/CO	S/CO	S/CO	S/CO	S/CO
**Sample A**							
Neat	16						
10	*14.37*	*5.14*	*5.14*	*13.03*	*13.87*	*10.49*	*8.93*
20	*11.91*	*3.62*	*2.87*	*11.89*	*11.59*	*7.65*	*6.45*
40	*10.04*	*1.64*	*1.66*	*10.49*	*8.62*	*5.46*	*3.69*
60	*7.83*	*1.23*	*1.15*	*8.45*	*7.00*	*3.65*	*2.44*
100	*5.64*	0.69	0.68	*6.28*	*4.13*	*2.69*	*1.58*
200	*2.91*	0.36	0.33	*3.69*	*2.48*	*1.24*	0.94
500	*1.44*	0.15	0.14	*1.52*	0.98	0.41	0.39
1000	0.91	0.09	0.08	0.78	0.53	0.31	0.21
**Sample B**							
Neat	16.26						
10	*13.61*	*4.79*	*3.80*	*12.96*	*11.49*	*9.10*	*7.62*
20	*11.31*	*2.43*	*2.23*	*10.72*	*9.27*	*6.60*	*4.97*
40	*8.74*	*1.24*	*1.04*	*8.36*	*6.63*	*4.10*	*3.07*
60	*6.29*	0.90	0.72	*7.11*	*5.26*	*3.06*	*1.99*
100	*4.61*	0.55	0.45	*5.29*	*3.29*	*2.20*	*1.18*
200	*2.48*	0.27	0.24	3.14	*1.76*	*1.09*	0.64
500	*1.05*	0.13	0.11	1.39	0.76	0.48	0.34
1000	0.42	0.08	0.08	0.73	0.49	0.24	0.18

Each sample dilution was tested one time as 12 mm and 6 mm DBS eluted in elution buffer (EB) and assay diluent (AD) using anti-HCV standard and modified assay files. *STD* standard assay, *MSV* modified sample volume, *EB* elution buffer, *AD* assay diluent, *S/CO* signal to cutoff ratio. Italic values indicates reactive samples with S/CO ≥ 1.00.

**Figure 1:**
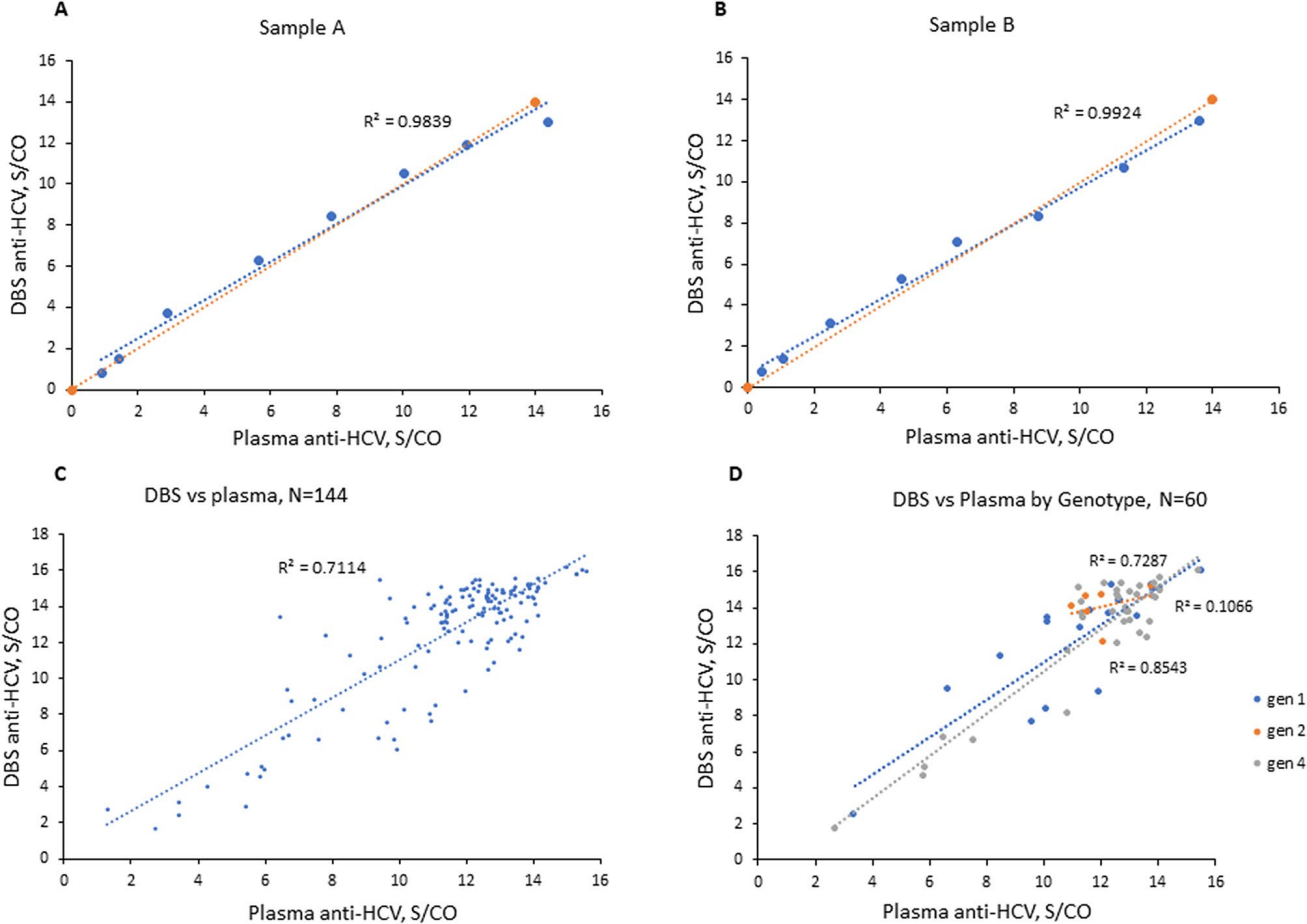
Comparison of DBS and plasma results. (**a**,**b**) anti-HCV S/CO values for DBS from dilution series are plotted against S/COs for matched plasma dilutions (R^2^ = 0.98, 0.99). Two highly reactive plasma samples A (genotype 1b) and B (genotype 1a) were serially diluted in plasma and whole blood at 8 dilution levels: 1:10, 1:20, 1:40, 1:60, 1:100, 1:200, 1:500 and 1:1000. 12-mm DBS eluted in EB were tested using the modified sample volume assay and plasma was tested using the standard anti-HCV assay. Blue dotted line shows linear regression; orange dotted line represents an equivalency line. (**c**) anti-HCV results obtained from matched DBS and plasma clinical samples (N = 144) tested with the modified and the standard assays, respectively (R^2^ = 0.71). (**d**) DBS anti-HCV S/COs plotted against plasma S/COs by genotypes. Series on the plot represent genotypes: 1 (n = 18), 2 (n = 6), 4 (n = 36); R^2^ = 0.73, 0.11, 0.85, respectively. Difference in DBS S/CO values between genotype groups is not significant (p > 0.05): genotype 1 vs 2, *p* = 0.055; 1 vs 4, *p* = 0.589; 2 vs 4, *p* = 0.08.

When DBS were eluted in assay diluent (AD), we observed a roughly two-fold decrease in assay sensitivity compared to elution buffer (EB). Modification of the assay file to increase the sample input resulted in a near ten-fold higher sensitivity for 12-mm DBS eluted in EB (1:500 vs 1:60, Table 1). Dilutional sensitivity using the modified assay file with 12-mm DBS eluted in EB was equivalent to the standard on-market assay with plasma samples; both DBS and plasma were positive at a dilution of 1:500 with comparable S/CO values (Table 1). Dilutional sensitivity decreased nearly 2.5-fold with the use of 6-mm DBS; the 6-mm DBS were reactive at a 1:200 dilution compared to plasma at a 1:500 dilution. Based on these optimization results, 12-mm DBS eluted in EB were used for all subsequent experiments. Results for the dilution series using 12-mm DBS with the modified input volume assay showed a strong correlation (R^2^ = 0.98, 0.99) between DBS and plasma results (Fig. 1a,b).

### Assay precision using DBS samples

Assay precision was determined using DBS from negative whole blood and a panel of low, middle, and high positive DBS samples. The DBS panel was prepared by diluting anti-HCV positive plasma spiked into negative whole blood and diluted at 1:20, 1:100, 1:200, and 1:500. The DBS samples were tested in 3 replicates per run, in 3 separate runs, on 2 ARCHITECT *i*2000SR instruments. Precision data are summarized in Table 2. The total coefficient of variation (CV) was < 20% for negative DBS, < 15% for low positive DBS, and < 10% for middle and high positive DBS samples.

**Table 2:** Precision of the modified sample volume ARCHITECT anti-HCV assay using whole blood DBS samples.

Instrument	Run	DBS Rep	Neg whole blood	Sample A dilutions			
				D 1:500	D 1:200	D 1:100	D 1:20
				Low Pos -1	Low Pos-2	Mid Pos	High Pos
1	1	1	0.09	2.11	4.15	6.69	12.10
1	1	2	0.09	2.23	4.16	6.56	11.60
1	1	3	0.04	1.63	4.17	6.57	12.28
1	2	1	0.10	2.03	4.28	6.61	11.50
1	2	2	0.09	1.79	3.33	5.42	11.23
1	2	3	0.09	1.84	4.31	6.32	11.27
1	3	1	0.08	1.39	4.07	6.58	11.98
1	3	2	0.09	1.95	3.99	6.59	8.44
1	3	3	0.10	2.03	4.33	4.84	11.15
Mean			0.09	1.89	4.09	6.24	11.28
SD			0.02	0.26	0.31	0.65	1.14
%CV			21.16	13.71	7.47	10.49	10.10
2	1	1	0.10	2.38	4.45	7.00	13.07
2	1	2	0.10	2.26	4.29	7.13	13.22
2	1	3	0.09	2.18	4.48	6.72	13.40
2	2	1	0.10	2.23	4.89	7.38	13.01
2	2	2	0.11	2.34	4.41	6.94	12.75
2	2	3	0.11	2.21	4.50	6.79	12.54
2	3	1	0.11	2.12	4.79	7.43	12.63
2	3	2	0.11	2.02	4.79	6.62	13.01
2	3	3	0.10	1.98	4.62	7.21	13.15
Mean			0.10	2.19	4.58	7.02	12.98
SD			0.01	0.13	0.20	0.29	0.28
CV%			6.84	6.11	4.45	4.07	2.18
Total			N = 18				
Mean			0.09	2.04	4.33	6.63	12.13
SD			0.02	0.25	0.36	0.63	1.19
%CV			17.12	12.42	8.24	9.56	9.78

*SD* standard deviation.

### Concordance with plasma results (clinical sensitivity) and specificity

**Figure 2:**
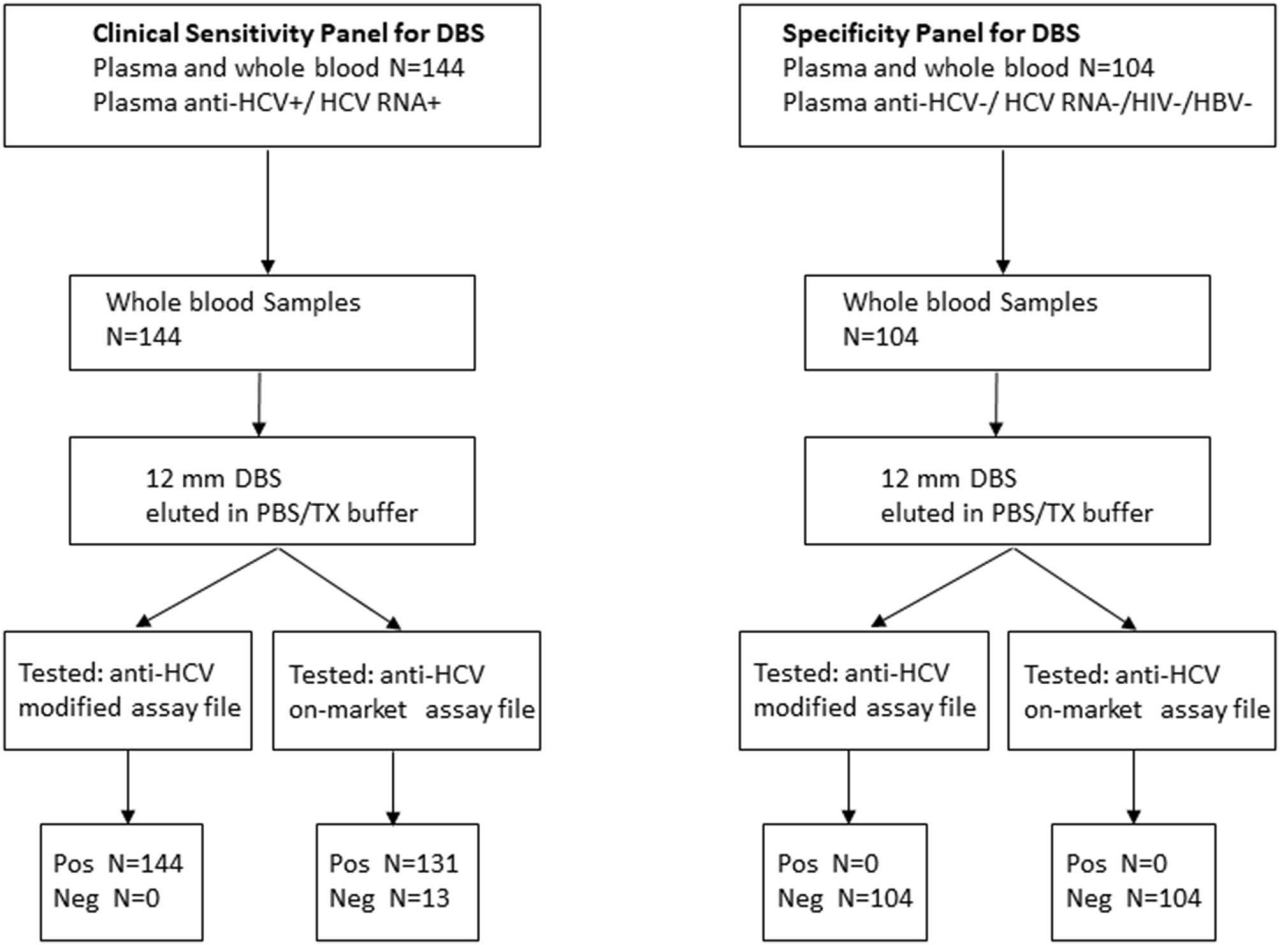
Flow chart for testing DBS samples to evaluate clinical sensitivity and specificity of the anti-HCV assays.

For N = 144 anti-HCV/RNA positive matched samples, DBS samples were prepared and tested using the standard and modified anti-HCV assay (Fig. 2), and the results were compared to those from plasma samples. The DBS and plasma results including range, median, and standard deviation are summarized in Table 3. All 144 DBS were positive with the modified assay while 131DBS samples were positive with the standard assay Correlation between DBS and plasma anti-HCV results is shown in Fig. 1c (N = 144, R^2^ = 0.71). Results were highly correlated for genotypes 1 and 4 with R^2^ > 0.7 (Fig. 1d; gen-1 n = 18, R^2^ = 0.73; gen-4 n = 36, R^2^ = 0.85). Genotype 2 had limited number of samples (N = 6) with S/CO range of 11.00–13.81 for plasma and 12.09–15.19 for DBS (R^2^ = 0.11). The difference in DBS S/CO values was not significant (p > 0.05) between genotype groups (gen 1 vs 2, *p* = 0.055; gen 1 vs 4, *p* = 0.589; gen 2 vs 4, *p* = 0.080).

**Table 3:** Summary of DBS anti-HCV results for matched clinical samples (N = 144).

	Plasma viral load, log IU/ml	Plasma anti-HCV, S/CO	DBS anti-HCV STD assay, S/CO	DBS anti-HCV MSV assay, S/CO
Range	< 1.48–6.42	1.28–15.55	0.2–16.38	1.67–16.21
Median	4.74	12.16	8.27	13.81
SD	0.97	2.70	4.82	3.36
Positive, N (%)	144	144	131 (90.97%)	144 (100%)

Viral load results detected < 1.48 log IU/ml for three samples were assigned value of 1.40 log IU/ml for calculation of median and standard deviation values. DBS were tested using standard (STD) and modified sample volume (MSV) assays. *SD* standard deviation.

Performance of the DBS assays was evaluated using accuracy characteristics as sensitivity, specificity, predictive values and likelihood ratios (LR)^12^. Agreement between DBS test results and the reference standard plasma test, and calculations are shown in Table 4.

**Table 4:** Diagnostic testing accuracy characteristics.

	Disease present	Disease not present	Total
	Plasma anti-HCV+	Plasma anti-HCV−	
**DBS test result**	**Anti-HCV modified assay file**		
Positive	144 (TP)	0 (FP)	144 (TP + FP)
Negative	0 (FN)	104 (TN)	104 (TN + FN)
Total	144 (TP + FN)	104 (TN + FP)	248
Sensitivity = TP/(TP + FN) = 144/144 × 100% = 100% (95% CI 97.47–100.00)			
Specificity = TN/(TN + FP) = 104/104 × 100% = 100% (95% CI 96.52–100.00)			
Positive predictive value (PPV) = TP/(TP + FP) = 144/144 × 100% = 100% (95% CI 97.40–100.00), calculated at 58.06% prevalence (144 positive/248 total)			
Negative predictive value (NPV) = TN/(TN + FN) = 104/104 × 100% = 100% (95% CI 96.44–100), calculated at 58.06% prevalence			
Positive likelihood ratio (LR+) = sensitivity/(1-specificity) = NA			
Negative likelihood ratio (LR−) = (1-sensitivity)/specificity = 0 Area under the ROC curve (AUC) = 1.00			
**DBS test result**	**Anti-HCV on-market assay file**		
Positive	131 (TP)	0 (FP)	131 (TP + FP)
Negative	13 (FN)	104 (TN)	117 (TN + FN)
Total	144 (TP + FN)	104 (TN + FP)	248
Sensitivity = TP/(TP + FN) = 131/(131 + 13) × 100% = 90.97% (95% CI 85.06–95.11)			
Specificity = TN/(TN + FP) = 104/104 × 100% = 100% (95% CI 96.52–100.00)			
Positive predictive value (PPV) = TP/(TP + FP) = 131/131 × 100% = 100% (95% CI 97.22–100.00), calculated at 58.06% prevalence			
Negative predictive value (NPV) = TN/(TN + FN) = 104/(104 + 13) = 88.89% (95% CI 81.75–93.95), calculated at 58.06% prevalence			
Positive likelihood ratio (LR+) = sensitivity/(1-specificity) = NA			
Negative likelihood ratio (LR−) = (1-sensitivity)/specificity = (1–0.9097)/1 = 0.09 (95% CI 0.05–0.15) Area under the ROC curve (AUC) = 1.00			

*TP* true positive, *FN* false negative, *FP* false positive, *TN* true negative, *NA* not applicable.

The sensitivity of the modified assay and on-market assay with DBS as a sample was 100% and 90.97%, respectively. Specificity of the DBS assay evaluated using whole blood from 104 anti-HCV negative samples (Fig. 2) was 100% for both assays (Table 4). The mean DBS S/CO value was 0.11 (95% CI 0.10–0.12) for the modified and 0.032 (95% CI 0.030–0.035) for the on-market assay. Positive predictive value (PPV) was 100% for both assays. Negative predictive value (NPV) was lower for the on-market assay: 88.89% vs 100% for the modified assay. Receiver operating characteristic (ROC) plots were very similar for both assays (Supplemental Fig. 1), with area under the ROC curve (AUC = 1.00) indicating a “perfect assay”.

### DBS stability

Anti-HCV stability in DBS samples was evaluated on DBS stored at − 20 °C, room temperature (RT) and + 37 °C for 2 weeks and tested for anti-HCV on day 1, 3, 7 and 14. DBS samples remained stable with variability in S/CO values relative to day-1 ≤ 10% at all storage conditions (Supplemental Table 1).

## Discussion

We optimized the commercially available ARCHITECT Anti-HCV assay for the detection of anti-HCV antibody in DBS samples prepared from venous whole blood to ensure high sensitivity and achieve equivalent performance to the assay run with venous plasma samples. The best dilutional sensitivity was achieved with 12-mm DBS eluted in PBS/Triton buffer and tested using a modified assay file with the increased sample input volume. The dilutional sensitivity using the modified assay file for DBS was equivalent to that of the commercially available assay for plasma and serum samples. We also demonstrated equivalency of assay results from DBS and venous plasma with dilution series and with 144 paired venous plasma and whole blood clinical specimens. The equivalency of ARCHITECT Anti-HCV test results for the 144 DBS and venous plasma samples, defined as the mean ratio of DBS S/CO to plasma S/CO, was 1.10 (95% CI 1.07–1.14) for the modified assay and 0.67 (95% CI 0.61–0.73) for the standard assay. While anti-HCV could be detected in DBS without modifying the assay file, increasing the sample input volume resulted in a nearly tenfold higher sensitivity for 12-mm DBS eluted in PBS/Triton buffer. Assay sensitivity was lower for 6-mm DBS samples and for DBS samples eluted in an alternative elution buffer (assay diluent).

Previous studies have evaluated DBS as a sample type for existing anti-HCV assays using various blood sample volumes, elution volumes, elution buffers, and assay volumes^13–15^. One study comparing more than 339 paired serum and DBS whole blood samples demonstrated 97.8% sensitivity and 100% specificity (100 µl sample, 1 ml elution volume, and 20 µl sample input volume)^14^. Another study including 511 patient samples reported 99.1% sensitivity and 98.2% specificity for the ARCHITECT Anti-HCV assay using DBS prepared from 50 µl venous whole blood, eluted with 1 ml buffer, and run using a 20 µl sample input volume^15^. Several other studies have reported relatively high sensitivity and specificity for matched venous plasma/serum and DBS samples^9,16–18^. Good assay performance has also been reported for anti-HCV assays using DBS samples from patients with HIV/HCV co-infection^11,19^.

Our findings suggest that modification of the ARCHITECT Anti-HCV assay input volume improved accuracy when using DBS compared to the standard assay volume, with high clinical sensitivity (100%) and specificity (100%) and concordant results with venous plasma samples.

We found that the ARCHITECT Anti-HCV assay and reagents, when used with a modified instrument pipetting algorithm, can accurately detect HCV antibodies in DBS samples of venous whole blood, yielding results that are comparable to those with venous plasma. This is an important finding because it shows equivalency of anti-HCV results from DBS and plasma samples and can be used to design future DBS studies with better sensitivity, which has important implications for expanding access to HCV testing. DBS samples are stable at room temperature (ST1), which simplifies storage and transportation of samples to central labs for testing. In addition, capillary blood collection removes the need for trained phlebotomists to conduct venipuncture, thereby extending state-of-the-art HCV diagnostic testing to underserved or marginalized populations with low access to direct HCV care, such as homeless persons, people who inject drugs, and those living in difficult-to-reach remote or rural areas.

Of note, our study included a limited number of samples collected from adults in a single country, and DBS were generated using venous whole blood, rather than fingerstick capillary blood. Sensitivity and specificity will need to be verified in larger and geographically diverse populations, including children, as well as in real-world field testing. Future studies will also need to directly compare assay performance with matched venous whole blood and capillary blood DBS samples.

## Methods

All methods in the study were performed in accordance with the guidelines of the The World Medical Association (WMA) Declaration of Helsinki.

### Samples

The research collaborative study was a retrospective study with convenience sampling. Matched venous plasma and whole blood specimens were collected from individuals in Cameroon in 2007–2015 after obtaining informed consent. All specimens were de-identified after collection. The collaborative study was approved by the Faculty of Medicine and Biomedical Science IRB and the Ministry of Health (MoH) in Cameroon, and by the Cameroon National Ethical Review Board. Plasma samples were pre-screened for HCV RNA as described previously^20^. HCV genotypes 1, 2, and 4 were present in the study population. HCV RNA-positive plasma samples were tested for HCV antibody; a subset of 144 matched pairs of venous plasma/whole blood samples positive for both HCV RNA and HCV antibody was selected for this DBS study (Fig. 2, Table 3). Of these 144 samples, 60 had available genotype classification: genotype 1 (30%), 2 (10%), and 4 (60%). Whole blood samples (N = 104) with matched plasma prescreened and negative for HIV, HBV, and HCV RNA and antibody were used to evaluate the specificity of the DBS assay (Fig. 2). HCV antibody-positive plasma samples A (genotype 1b, 16 S/CO) and B (genotype 1a, 16.26 S/CO) serially diluted in normal human plasma and normal whole blood were used to evaluate dilutional sensitivity and assay precision. DBS samples were prepared by spotting 70 µl of whole blood on a perforated circle of a Whatman 903 DBS card (GE Healthcare, Little Chalfont, UK) and dried overnight at room temperature.

### DBS anti-HCV testing

After drying overnight, individual 12-mm DBS were punched from the perforated cards and inserted into individual Eppendorf microtubes with 350 µl elution buffer (EB; PBS pH 7.4, 0.25% Triton X-100) or alternative buffer (AD; assay diluent from Abbott ARCHITECT anti-HCV reagent kit). The tubes were incubated at room temperature on a shaker for 1 h and the eluate was transferred into a test tube, centrifuged at 10,000 RCF for 5 min, and placed on the ARCHITECT *i*2000SR analyzer for testing using the Abbott ARCHITECT Anti-HCV assay. The assay file was modified to allow a maximum 150 µl sample input volume to use with DBS. To compare relative sensitivities of the assay using 12-mm to 6-mm DBS, both DBS sizes were prepared from the same sample and processed in an identical manner. The 6-mm and 12-mm DBS were eluted in either EB or AD and tested using the standard assay or the modified sample input volume. 12-mm DBS from the sensitivity and specificity panels eluted in EB were tested with both assays to evaluate clinical sensitivity and specificity (Fig. 2). Venous plasma samples were tested with the standard ARCHITECT Anti-HCV assay according to the package insert^20^. Assay results for DBS and plasma samples were compared.

The Abbott ARCHITECT Anti-HCV assay^21^ is an automated two-step chemiluminescent microparticle immunoassay (CMIA) for the qualitative detection of antibody to hepatitis C virus (anti-HCV) in human serum and plasma. A chemiluminescent signal is measured as relative light units (RLUs). The presence of anti-HCV is determined by comparing the sample relative light units (RLU) to the calibrator cutoff RLU (S/CO). The specimen is considered reactive for anti-HCV if the S/CO is ≥ 1.00.

### Stability testing

DBS spotted in replicates from three whole blood samples were used to evaluate the stability of anti-HCV in DBS samples. The first testing was done on day 1 after drying the DBS samples at room temperature overnight; each sample was tested in triplicates, and the mean S/CO was used as the baseline result for each sample. The DBS samples were stored individually in plastic bags with desiccant at − 20 °C, room temperature (RT) and + 37 °C for 2 weeks and were tested for anti-HCV on days 3, 7 and 14. The differences in S/CO values compared to the day 1 baseline mean are reported in the Supplemental Table 1.

### Statistical analysis

Data sets were summarized to report the n value, range, mean or median, standard deviation, CV%, 95% confidence interval. Descriptive statistics and R^2^ were calculated using Excel for Microsoft 365 version 2102; genotype group comparisons were calculated using a two-tailed t-test, statistical significance was considered met if *p* < 0.05. R 3.5.3 software was used to produce the ROC plots and calculate the AUC.

## Supplementary Information

Supplementary Information.

## Data Availability

The datasets generated during and/or analyzed during the current study are available from the corresponding author on reasonable request.
