# Supplementary material for: Evaluation of hepatitis C virus antibody assay using dried blood spot samples
Source: Sci Rep. 2022 Mar 8;12:3763. doi: 10.1038/s41598-022-07821-0 (PMC8904514; doi:10.1038/s41598-022-07821-0)
Supplement: Supplementary file 1 — Supplementary Information. [file 41598_2022_7821_MOESM1_ESM.pdf]

Evaluation of hepatitis C virus antibody assay using dried blood spot samples

Vera Holzmayer[1*], Russell Taylor[1], Mary C Kuhns[1], Susan H Gawel[1], Nicaise Ndembi[2], Dora Mbanya[3], Lazare Kaptue[4†], Mary A Rodgers[1], Gavin Cloherty[1]

[1] Abbott Laboratories, Abbott Diagnostics Division, Abbott Park, Illinois

[2] Africa Centres for Disease Control and Prevention, Addis Ababa, Ethiopia

[3] Universite de Yaounde I, Yaounde, Cameroon

[4] Universite des Montagnes, Bangangte, Cameroon

*Corresponding author: Vera Holzmayer

100 Abbott Park Road, Bldg. AP20

Abbott Park, IL 60064-3500

Email: vera.holzmayer@abbott.com

Phone: 224-668-0756

† In memoriam.

Supplemental Table 1. Anti-HCV Results for DBS Stored at Various Times and Temperatures.

Day	-20°C	RT	+37°C	-20°C	RT	+37°C
	**Sample 1, DBS S/CO**			**S/CO change from Day 1, %**		
1		9.52				
1		9.1				
1		9.47				
1	**mean**	**9.36**				
3	9.16	10.07	9.89	-2.1	7.6	5.7
7	10.24	10.02	9.46	9.4	7.1	1.1
14	9.72	9.07	8.41	3.8	-3.1	-10.1
	**Sample 2, DBS S/CO**			**S/CO change from Day 1, %**		
1		12.85				
1		12.49				
1		12.61				
1	**mean**	**12.65**				
3	12.74	12.96	12.29	0.7	2.5	-2.8
7	12.69	12.25	12.43	0.3	-3.2	-1.7
14	12.78	12.07	11.58	1.0	-4.6	-8.5
	**Sample 3, DBS S/CO**			**S/CO change from Day 1, %**		
1		14.54				
1		14.23				
1		14.62				
1	**mean**	**14.46**				
3	14.49	14.65	14.27	0.2	1.3	-1.3
7	14.64	14.56	13.94	1.2	0.7	-3.6
14	14.86	14.11	13.88	2.8	-2.4	-4.0

Anti-HCV DBS stability testing was done using three whole blood samples. DBS were spotted on Day 0, dried overnight at room temperature (RT) and tested for anti-HCV the next day (Day 1) in 3 replicates. DBS were stored for 2 weeks at -20°C, RT and +37°C, and tested for anti-HCV on day 3, 7 and 14. S/COs for each test and changes (%) from day 1 are shown in the table. The maximum change of -10.1% was observed for sample 1 stored at +37°C. DBS stored at RT had < 5% change from day 1.

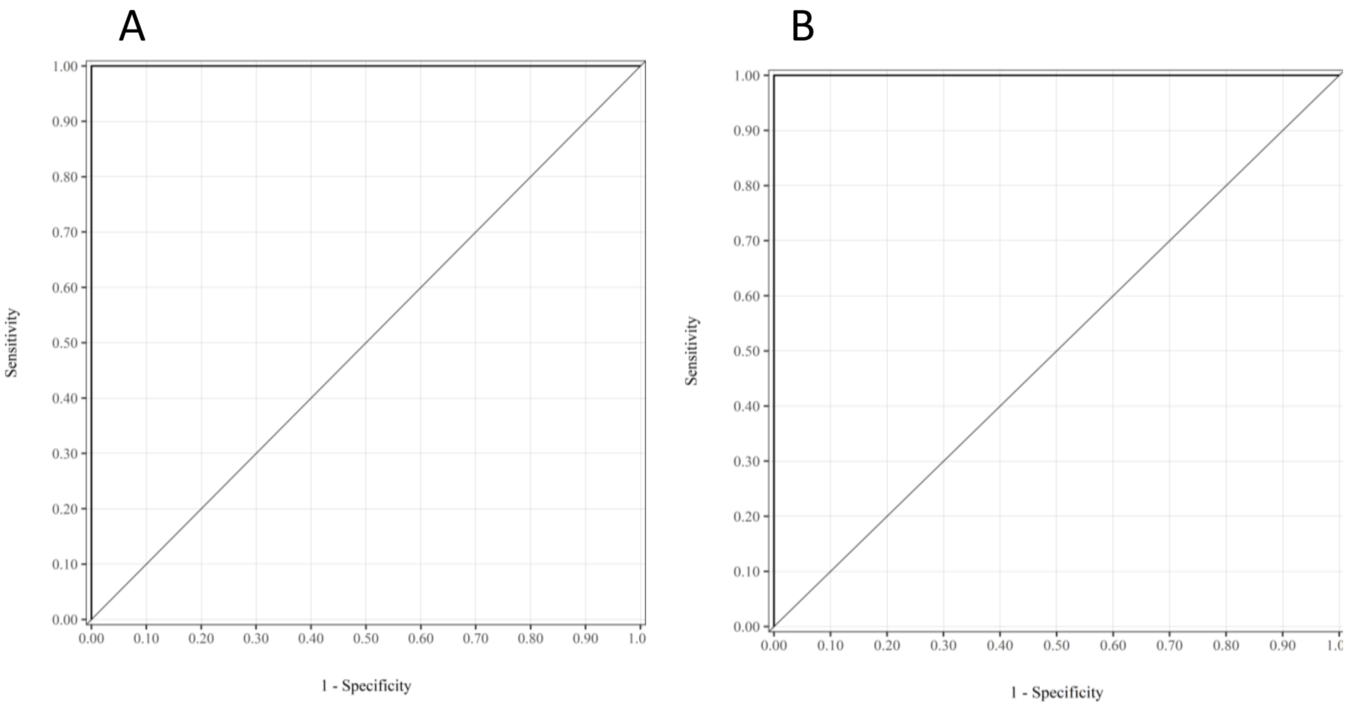

Supplemental Figure 1. Receiver operating characteristic (ROC) plots for various cutoff levels for DBS samples tested using anti-HCV on-market (A) and investigational assay (B). Total number of DBS samples tested N=248: 144 plasma anti-HCV positive and 104 plasma anti-HCV negative. Sensitivity is plotted vs (1-Specificity).
